# Novel Ruthenium-Silver PTA-Based Polymers and Their Behavior in Water

**DOI:** 10.3390/polym11081249

**Published:** 2019-07-28

**Authors:** Benjamin Sierra-Martin, Manuel Serrano-Ruiz, Franco Scalambra, Antonio Fernandez-Barbero, Antonio Romerosa

**Affiliations:** 1 Department of Chemistry and Physics, University of Almeria, 04120 Almeria, Spain; 2 Inorganic Chemistry Lab-CIESOL, Department of Chemistry and Physics, University of Almeria, 04120 Almeria, Spain; 3 Institute of Applied Chemical Sciences, Universidad Autonoma de Chile, 8320000 Santiago, Chile

**Keywords:** organometallic, ruthenium complexes, PTA, polymer swelling, light scattering

## Abstract

New coordination polymers based on two metal-containing moieties Ru–Ag are synthesized: Na[RuCpX(PTA)-μ-(PTA)-1κ*P*:2κ^2^*N*-AgX_2_]_∞_ (X = Cl (**1**), Br (**2**), I (**3**)). Characterization is performed by NMR, UV-visible and FT-IR spectroscopy, optical-electron microscopy, and elemental analyses (C, H, N, S). Light scattering is employed to characterize the colloidal particles growth by polymer self-assembling. These structures are stable over a broad range of pH and exhibit thermally-driven swelling, thus resembling a typical thermosensitive hydrogel.

## 1. Introduction

The coordination chemistry of metal complexes containing 1,3,5 triaza-7-phosphaadamantane (PTA) has received great interest in recent years. The attractiveness of such ligand arises from its strong binding capability as well as its unusual solubility in water. PTA ligands traditionally coordinate metals through the phosphorus atom. During last years, bounding of PTA ligands to boron or transition metals through the nitrogen atoms has been explored and as a consequence of that, a new family of metal–organic polymers in which PTA ligands link the metal centers through both phosphorus and nitrogen atoms was synthetized [1,2].

In 2005, the first example of metal-backbone polymer containing the PTA ligand for [{CpRu(PTA)_2_(DMSO-k*S*)}{AgCl_2_}]_∞_ was reported, being also the first case of hydro-soluble polymer bearing organometallic units [1]. The complex consisted of {RuCp(DMSO-κ*S*)(PTA-κ*P*)}^+^ moieties linked each other by {AgCl_2_}^-^ anions through the N_PTA_. This strategy gave rise to Ru–Ag lineal heterometallic polymers with the PTA ligand bi-coordinated by P to Ru and by N to Ag, thus linking both metals. In order to widen this family, reactions of PTA-Ru containing complexes against different metal complexes were designed. The synthesis of a hydro-soluble Ru–Ru–Au polymer [{RuCp(PTA)_2_-μ-CN-1κ*C*:2κ^2^*N*-RuCp(PTA)_2_}-μ-Au(CN)_4_]_n_ (PTA = 1,3,5-triaza-7-phosphaadamantane) was reached, being the first example of a metal-organic polymer behaving as thermo-gel in water [3]. Additional synthesis were reckoned with the extended formulas [{RuCp(PTA)_2_-μ-CN-1κ*C*:2κ^2^*N*-RuCp(PTA)_2_}-μ-MX_m_]_n_ (M = transition metal; X = halide, pseudohalide), [{RuCp(PTA)_2_-μ-CN-RuCp(PTA)_2_}-μ-NiCl_3_]_n_ [4] and [{RuCp(PTA)_2_-μ-CN-RuCp(PTA)_2_}-μ-CdCl_3_]_n_ [5]. 

Complexes containing {Ru–CN–Ru}^+^ moieties manifests hydro-solubility and confinement of structural water, as recently reported for *trans*-[{RuCp(PTA)_2_-μ-CN-1κ*C*:2κ^2^*N*-RuCp(PTA)_2_}-μ-CoCl_3_]n(DMSO)_n_ and *cis*-{[{RuCp(PTA)_2_-μ-CN-1κ*C*:2κ^2^*N*-RuCp(PTA)_2_}-μ-CoCl_3_]}_n_{[RuCp(PTA)_2_-μ-CN-1κ*C*:2κ^2^*N*-RuCp(PTA)_2_]Cl}0.5_n_·(15H_2_O)_n_ polymers. As observed by neutron scattering, they are porous supramolecular systems where structural water molecules are confined into nano-channels [6]. Different features, including crystallinity, make of these polymers a new class of materials lying between metal organic frameworks (MOFs) and infinite coordination polymers (ICPs) [7]. It is worth mentioning that metal polymers containing PTA and PTA-derivatives show interesting catalytic and bactericide properties [8,9,10].

Nevertheless, despite of different unsuccessful attempts have been performed to obtain new heterometallic complexes from [{RuCpY(PTA)(PTA-κ*P,N*)}-μ-MX_m_]_n_, only one example has been obtained till now with heterometallic polymers [{RuCpL(PTA)(PTA-κP,N)}-μ-MX_m_]_n_ (M = transition metal; L= X, DMSO, H_2_O; X = halide, pseudohalide). The goal of the present paper is to report a new strategy of synthesis that overcomes the previous difficulties and is able to lead to three new heterometallic polymers. They are constituted by a {RuCpX(PTA)_2_}^+^ moiety linked by {AgX_2_}^-^ units (X = Cl, Br, I). We will also show that these systems self-assemble to form mesoscopic hydrogel particles with thermal-sensitive structure.

## 2. Materials and Methods

### 2.1. Materials

All chemicals were reagent grade and (unless otherwise stated) used as received by commercial suppliers. Reactions were carried out under pure argon atmosphere. Standard Schlenk-tube techniques with freshly distilled and oxygen-free solvents were employed. The hydrosoluble phosphine PTA and complex [RuClCp(PPh_3_)_2_] were prepared as reported in the literature [11,12].

### 2.2. Methods

^1^H and ^13^C{^1^H} NMR spectra were recorded on a Bruker DRX300 spectrometer (Billerica, MA, USA) operating at 300.13 MHz (^1^H) and 75.47 (^13^C), respectively. Peaks positions were relative to tetramethylsilane and calibrated against the residual solvent resonance (^1^H) or the deuterated solvent multiplet (^13^C). The same instrument operating at 121.49 is employed to get the ^31^P{^1^H} spectrum. Chemical shifts were relative to external 85% H_3_PO_4_ for ^31^P{^1^H} NMR with downfield values taken as positive. Spectra were always recorded at 298K and at a polymer concentration slightly below the solubility limit. Infrared spectra were determined as KBr disks using a ThermoNicolet Avatar 360 FT-IR spectrometer (ThermoFisher, Waltham, MA, USA). Elemental analyses (C, H, N, S) were performed on a Fisons EA 1108 elemental analyzer (Fison Instruments Ltd, Glasgow, UK). Dynamic Light Scattering (Malvern Instruments, Worcestershire, UK) was used to explore the colloidal scale of the self-assembled polymer units. A goniometer was equipped with a 5 mW He–Ne vertical-polarized laser (λ = 632.8 nm) as illumination source. A high efficiency photomultiplier performed the scattered photon harvesting at a 40° angle. The output signal fed an electronic setup to construct the auto-correlation function in real time and diffusion coefficients were extracted from the relaxation curve. Diffusion coefficients were finally converted into mean particle size by the Stokes–Einstein equation for spherical particles, once corrected by temperature and solvent viscosity [13]. Temperature was controlled within ± 0.1 K precision by a Peltier device coupled to a bath chamber. Scattering from supra-structure contributions and multiple scattering were minimized by working under diluted regime.

## 3. Results and Discussion

### 3.1. Synthesis of the Coordination Polymers

#### 3.1.1. Synthesis of System-1): Na[RuCpCl(PTA)-μ-(PTA)-1κP:2κ^2^N-AgCl_2_]n

**Figure 1:**
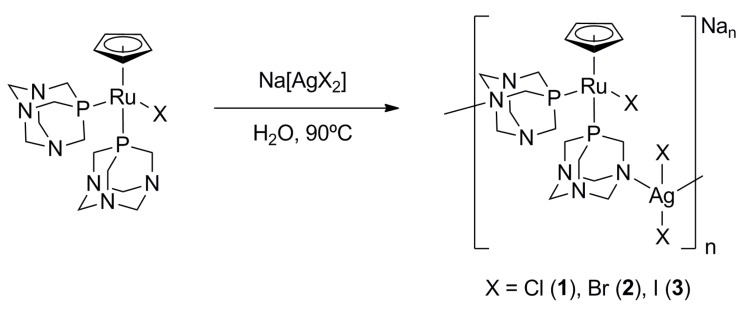
Synthesis scheme of polymers: system-**1**, system-**2** and system-**3**.

The Ru–Ag heterometallic polymers were synthesized by reaction of the [RuCpX(PTA)_2_] complex with AgOTf and NaX in water (Figure 1). The complex [RuCpCl(PTA)_2_] (43 mg, 0.08 mmol) was added (in the dark) to a solution of Na[AgCl_2_] (16.8 mg, 0.08 mmol), which was prepared in situ by reaction of AgOTf (20 mg, 0.08 mmol) and NaCl (1.0 mg, 0.17 mmol) in 2 mL of water at 90 °C. The solution was then cooled at room temperature after 10 min, filtered through a sintered glass filter and evaporated to 1 mL. It was dialyzed for several 5-min cycles against 500 mL of water at 22 °C, until total removal of Cl^-^. The solution was then evaporated before addition of 6 mL of ether: ethanol (1:1) and sonication for 10 min. The powder obtained after filtration was finally dried under vacuum.

Yield: 15 mg, 26.2%. Elemental analysis calculated for C_17_H_29_Cl_3_N_6_P_2_NaRuAg (717.7): C 28.45, H 4.07, N 11.71%; found: C 28.95, H 4.71, N 11.01%. S_25 °C_ (mg/cm^3^): 40. ^1^H NMR(300,13 MHz, D_2_O, 20 °C): δ (ppm) 3.95–4.10 (m, P-CH_2_(PTA), 12H), 4.48 (bs, N-CH_2_(PTA), 12H), 4.69 (bs, C_5_H_5_, 5H); Dept{^1^H} RMN (293 K, D_2_O, 75,467 MHz): δ (ppm) 54.15 (t, ^1^*J*_CP_ = 8.72 Hz, C(PTA)-P), 70.66 (s, C(PTA)-N, 12H), 77.79 (s, C_5_H_5_). ^31^P{^1^H} NMR (121.49 Mz, D_2_O, 20 °C): *δ* (ppm) –25.65 (s, PTA). 

#### 3.1.2. Synthesis of System-2 and System-3: Na[RuCpBr(PTA)-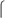-(PTA)-1|P:2|^2^N-AgBr_2_]_n_ (System-2), Na[RuCpI(PTA)-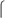-(PTA)-1|P:2|^2^N-AgI_2_]_n_ (System-3)

Similar procedure was used for the rest of heterometallic halogens: Br and I. The initial complexes (with the selected halogen) were obtained by the reaction (in the presence of 2 mL of water) of [RuCpCl(PTA)_2_] (43 mg, 0.08 mmol) with 1.4 eq. of NaBr (8.5 mg, 0.08 mmol) for System-**2** and NaI (12.5 mg, 0.08 mmol) for System-**3**, leading to [RuCpBr(PTA)_2_] and [RuCp(I)(PTA)_2_], respectively. The solutions containing the complexes were reacted at 90 °C with solutions obtained by mixing AgOTf (20 mg, 0,08 mmol) in 0.5 mL of water with 1.5 mL of an aqueous solution of NaBr (17.5 mg, 0.17 mmol) for System-**2**, or NaI (25.5 mg, 0.17 mmol) for System-**3**. The solutions further processing was performed following the same procedure used for System-**1**.

Polymer **2**: Yield: 22 mg, 32.3%. Elemental analysis calculated for C_17_H_29_Br_3_N_6_P_2_NaRuAg (851.0): C 23.99 H 3.43, N 9.85%; found: C 24.47, H 3.42, N 9.42%. S_25 °C_ (mg/cm^3^): 30. ^1^H NMR(300.13 MHz, D_2_O, 20 °C): δ (ppm) 3.96 (ABX system, NCH_2_P, 6H), 4.47 (bs, NCH_2_N, 6H), 4.65 (s, Cp, 5H); ^13^C{^1^H} NMR (75.47 MHz, D_2_O, 20 °C): δ (ppm) 54.81 (t, NCH_2_P), 70.55 (t, ^3^*J*_CP_ = 3.0 Hz, NCH_2_N), 77.00 (t, ^3^*J*_CP_ = 1.8 Hz, Cp). ^31^P{^1^H} NMR (121.49 Mz, D_2_O, 20 °C): δ (ppm) –26.36 (s, PTA).

Polymer **3**: Yield: 32 mg, 40.3%. Elemental analysis calculated for C_17_H_29_I_3_N_6_P_2_NaRuAg (992.04): C 20.58, H 2.94, N 8.47%; found: C 21.03, H 3.31, N 8.71%. S_25 °C_ (mg/cm^3^): 10. ^1^H NMR (D_2_O): δ (ppm) 3.89–4.12 (m, CH_2_P(PTA), 12H), 4.47 (bs, CH_2_N(PTA), 12H), 4.76 (bs, Cp, 5 H). ^13^C{^1^H} NMR (D_2_O): δ (ppm) 56.36 (t, CH_2_P(PTA)), 70.62 (s, CH_2_N(PTA)), 77.65 (s, Cp). ^31^P{^1^H} NMR (D_2_O): δ (ppm) –28.50 (s, PTA). 

Dialysis was successfully used for polymer purification after checking the presence of NaX impurities at the complexes. NMR indicated that polymers in water were in equilibrium with their PTA starting complexes [RuCpX(PTA)_2_] [13,14] and likely with {AgX_2_}^-^ as any AgX precipitation was observed by addition of AgOTf to the dissolution. 

### 3.2. Chemical Characterization

In order to obtain a statistically relevant NMR signal, large recording times were needed. This fact suggests that the observed signals come from a small amount of starting complex in equilibrium with the polymer complexes. The NMR thus reveals that the polymers were in equilibrium with their PTA starting complexes in water: [RuCpX(PTA)_2_] with X = Cl for **1** [13], X = Br for **2** [12], and X = I for **3** [14]. This fact was also confirmed by NMR diffusion studies in D_2_O, which showed that the hydrodynamic radius of the NMR active species in solution were similar to that found for the monometallic complexes. The large solubility of these complexes in water (S_25 °C_ = 40 mg/cm^3^ for **1**; S_25 °C_ = 30 mg/cm^3^ for **2;** and S_25 °C_ = 10 mg/cm^3^ for **3**) was likely the vector that favored the equilibrium with the polymers. Additional experiments were performed and are discussed in the next sections, with the aim of further confirming the polymeric character. Those experiments confirmed that the complexes **1**, **2** and **3**, in solution, displayed properties consistent with a polymeric composition and are similar to those found for [{CpRu(PTA)_2_(DMSO-k*S*)}{AgCl_2_}]_∞_, the unique published example of this kind of heterometallic polymer [1]. These results support the idea that complexes **1**, **2** and **3** have to be constituted by a polymeric structure in which the [RuCpX(PTA)_2_] moieties are linked by {AgX_2_}^-^ units coordinate to the N_PTA_ atom of both PTA ligands (Figure 1).

### 3.3. Optical Properties

**Figure 2:**
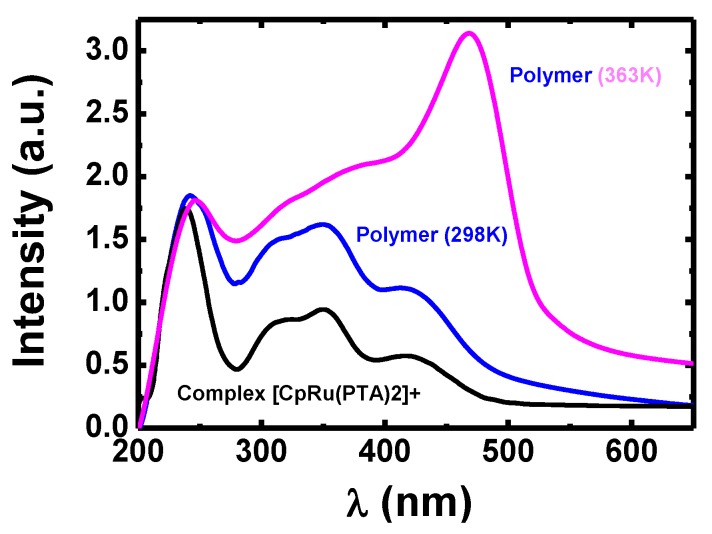
UV-Vis spectra for the monomer {RuCpCl(PTA)_2_}^+^ (black) and system-**1** at T = 298 K (blue) and T = 363 K (pink). Water at pH = 6.5 was used as reference solvent in all cases.

UV-Vis analysis was carried out at 298 K using a diode array spectrophotometer HR4000 (Ocean Optics, Largo, FL, USA) within the 200–1100 nm range. Figure 2 plots the spectra corresponding to the monomer unit {RuCpCl(PTA)_2_}^+^ and polymer system-**1**. The band at λ = 220 nm corresponds to the absorption of PTA. The Ru metal-to-ligand charge transfer transitions led to two absorbance bands located at λ = 350 nm and λ = 410 nm, which correspond to the Cp at lower energy. For the latter one, the increase of its extinction coefficient was due to the loss of structured water around the Cp at high temperature, with subsequent assembly of the polymer [15]. A similar situation would happen when temperature increased enough to break the structured water network observed at room temperature. This behavior agrees with the slight bathochromic shift observed for the complexes in organic solvents [16]. 

### 3.4. Non-Ionic Character of the Polymer

**Figure 3:**
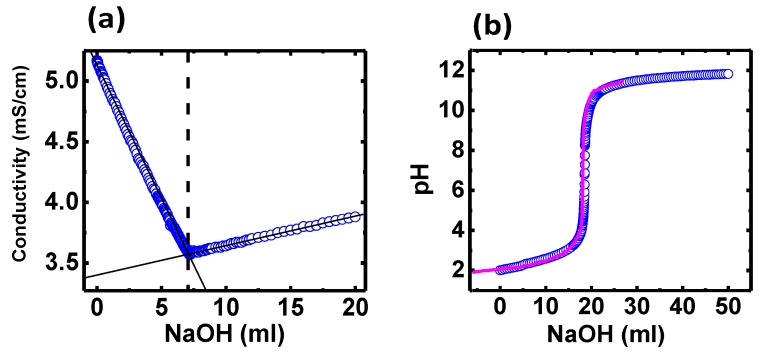
(**a**) Conductimetric and (**b**) potentiometric titrations of system-1. The sample was initially set up at pH = 2 by addition of HCl and afterwards it was titrated with NaOH. The decrease in conductivity in (**a**) was due to the neutralization of the excess of H+, while the subsequent increase arose from the excess of OH- added. Concomitantly, the pH of the complex solution was changing, as shown in (**b**), in the same way as a reference solution (pink line). Note that no region was observed between these linear trends in conductivity, so the polymer did not ionize at any pH.

Conductimetric and potentiometric titrations are carried out to investigate the ionization (charge-pH relation) of system-**1**. Both titrations were performed simultaneously by using a common cell for the pH and conductivity electrodes as well as for a nitrogen inlet tube. A 1 μL sensitivity dispenser was used for fluid injection. Gentle stirring diminished temperature and concentration gradients. The two regions in Figure 3a show only the neutralization of H^+^ ions and the excess of OH– ions, which indicated that any group in the complex can be ionized. Further, Figure 3b reveals that the ionization does not occur in the range of pH (2–12). Dyson et al. reported that PTA cannot be bis-protonated still under strong acid conditions [17]. In addition, calculations showed that the basicity of the ligand was significantly reduced when PTA was bonded to a ruthenium. It is then reasonable to think that PTA should reduce its basicity when it is bis-coordinated (to a ruthenium by the P and to another metal through its N) forming the polymeric structure. This explains why PTA ligands are not protonated even at low pH, which supports the polymeric nature of the complex. 

### 3.5. Polymer Self-Assembling

**Figure 4:**
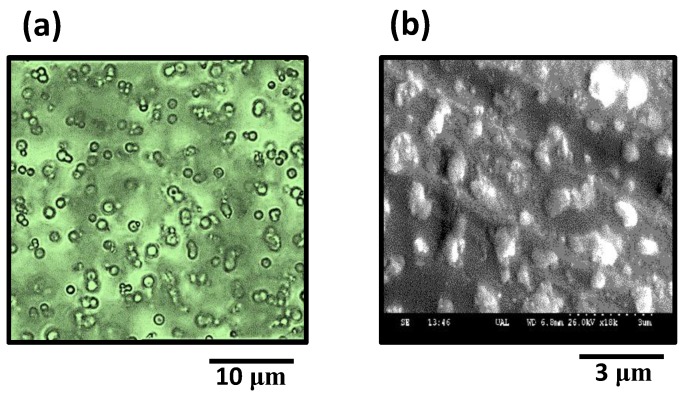
(**a**) Colloidal particles from the coordination polymer self-association (optical microscopy); (**b**) particles shape and heterogeneity as observed by scanning electron microscopy. Picture corresponds to the Ag–Cl polymer (System-**1).**

Polymer self-assembly was observed by direct optical and scanning electron microscopy. Optical observations were performed at 298 K with a Leica inverted transmission optical microscope (100 × magnification immersion ocular) coupled to a CCD camera. Figure 4a shows a scramble of spherical-shaped particles in water (shadows and steins around particles correspond to other particles located out of the focal plane, since non confocal microscopy was employed). Electron microscopy (Hitachi S-3500N, Hitachi High-Technologies, Krefeld, Germany) shows magnified dry polydisperse particles (Figure 4b), corresponding to the Ag–Cl polymer self-assembling (System-**1**). For sample preparation, a drop of polymer-water dispersion was deposited on a poly-L-Lysine coated slide in order to favor particles immobilization during the drying process. The metallic character of the coordination polymer allowed SEM observation without the aid of any additional metallic sputtering. Pictures show a polydisperse collectivity of ~2 μm averaged-size particles. 

### 3.6. Polymer De-Swelling

**Figure 5:**
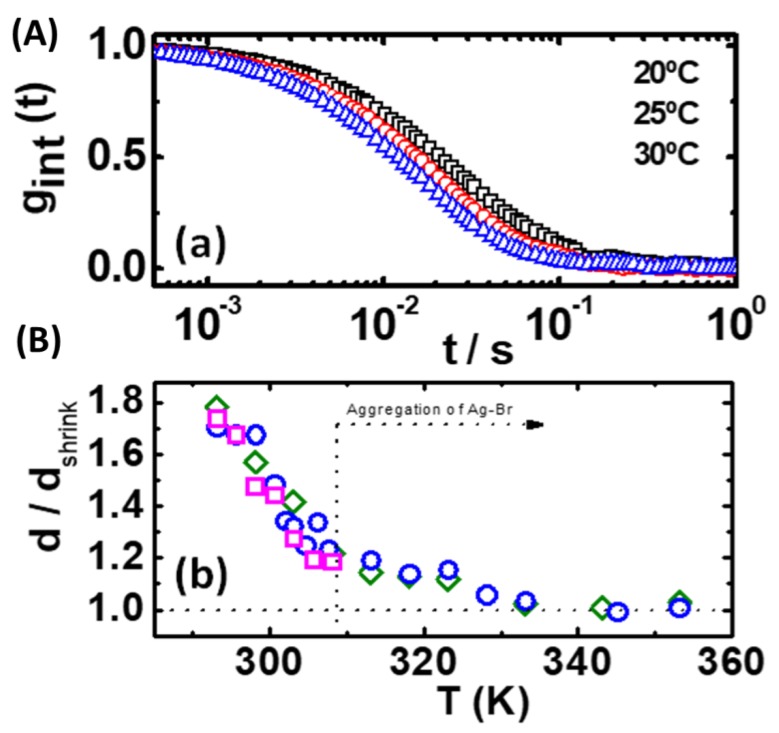
(**a**) Intensity autocorrelation function for the colloidal particles at different temperatures; (**b**) Temperature-controlled swelling for the three coordination polymer particles: **○** Ag–Cl, **□** Ag–Br, **◊** Ag–I.

Particle size of the polymer complexes was monitored by dynamic light scattering. Figure 5a shows the intensity autocorrelation function for Ru–Ag at different temperatures. Mean particle diffusion coefficients were calculated from the relaxation slopes and converted into mean particle size. Figure 5b shows the influence of the temperature on particle size for the three polymer systems, with identical behavior. Particle size decreased monotonically for increasing temperature until reaching a transition temperature at ~307 K. Beyond this point, particle size relaxed to reach a collapsed minimum size. This process was observed to be reversible over several temperature cycles, without any kind of hysteresis. The maximum swelling ratio was ~1.75, which means a 5.36-fold increase in particle volume.

Particles remained swollen at lower temperatures due to the high solubility of the PTA ligands. Water interacted strongly with the N_PTA_ atoms, forming an extensive hydrogen bond network. Additionally, water molecules were able to structure around the hydrophobic cyclopentadiene ligands, which produced an intensity reduction of the absorption coefficient of the Ru–Cp charge transfer [18]. We argue that the temperature dependence arises from the hydrophilic and hydrophobic character of the PTA and cyclopentadiene units, respectively. Below the transition temperature, particles were swollen because of the high solubility of PTA. Nitrogen atoms in PTA (N_PTA_) were bridged through extensive hydrogen bonding to the solvent water molecules [19]. In addition, water molecules formed clusters around the hydrophobic Cp ligands. Above the transition temperature, the water–amine hydrogen bonds were disrupted and the attractive hydrophobic interactions between the polymer chains became dominant; as a result, the polymer chains collapsed, expelling water from the particles [15].

**Figure 6:**
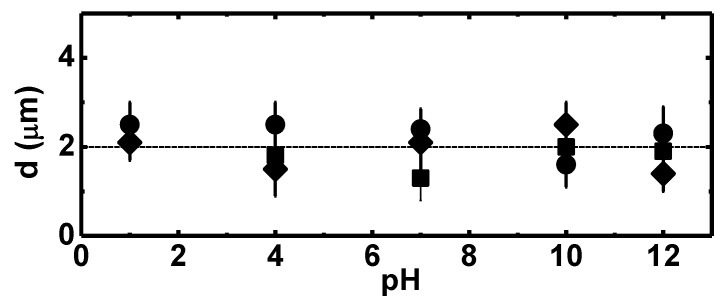
Influence of the pH on the self-assembled polymer at T = 298 K: ●Ag–Cl, ■ Ag–Br,♦ Ag–I.

The influence of the pH on the coordination polymer size is shown in Figure 6. The three systems under air atmosphere were stable along the whole pH range. Particle size remained constant due to the absence of ionized groups on the polymer chains, thus preventing extra swelling due to Donnan effect [20,21]. 

## 4. Conclusions

We have conducted the synthesis of three new coordination polymers based on two metal-containing moieties Ru–Ag bridged through the phosphine PTA (3,5,7-triaza-phosphaadamantane) with formula Na[RuCpX(PTA)-μ-(PTA)-1κ*P*:2κ^2^*N*-AgX_2_]_∞_ (X = Cl (**1**), Br (**2**), I (**3**)). These polymers self-associate in aqueous media to form stable micro-particles that interestingly exhibits a temperature-dependent size transition. The polymer particles are highly stable, without any particle aggregation over a broad range of pH. We think that swelling features described in this paper become a promising aspect leading to the design of effective temperature-tuned catalytic reactions.
